# Nursing Section

**Published:** 1904-01-02

**Authors:** 


### The Hospital.

### Nursing Section.

Contributions for this Section of "THE HOSPITAL" should be addressed to the EDITOR, "THE HOSPITAL" NURSING SECTION, 28 & 29 Southampton Street, Strand, London, W.C.

No. 901.—VOL. XXXV. *SATURDAY, JANUARY* 2, 1904.

### Notes on News from the Nursing World.

### A SPLENDID MEMORIAL.

THE final result of the appeal of the Women's Memorial to Queen Victoria and the Queen's Nurses Endowment Fund is, that the handsome sum of £84 000 has been added to the £72,000 of the Women's Jubilee Offering to Queen Victoria, making a total of £156,000 The amount collected by the central committee was made up as follows:—England, Wales, and the Colonies, £66,000; Scotland, £12,000; and Ireland, £6,000. But the most interesting feature is that about four million people subscribed to the Fund, and that, with the exception of one eighth of the amount contributed in sums of over £5, the bulk of the money was comprised of small sums ranging from one penny upwards. Both the King and Queen have expressed their gratification at the result of the appeal, and, bearing in mind the time it was issued, when the South African War was in progress and other very important appeals were straining the resources of the public, there can be no question as to the extremely satisfactory character of the response It shows that all classes rejoiced to avail themselves of the opportunity afforded them to manifest their affection for Queen Victoria by subscribing to the memorial which they felt that she herself would have approved. We think, too, that a portion of the credit is due to Lady Londonderry and the executive committee, whose skilful organising power and tireless energy were from the outset to the finish employed in order to bring the movement before the English-speaking community, and enlist the widest possible sympathy and support.

### HONOURS FOR MR. HAROLD BOULTON.

THE King has been graciously pleased to appoint to be member of the Fourth Class of the Royal Victorian Order Mr. Harold Edwin Boulton, honorary treasurer of Queen Victoria's Jubilee Institute for Nurses. This is a well-merited recognition by his Majesty of the indefatigable and valuable services of Mr. Boulton in the interests of the great movement for the promotion of district nursing.

### THE SCOTTISH BRANCH OF QUEEN VICTORIA'S JUBILEE INSTITUTE.

THE fifteenth annual report of the Scottish Branch of Queen Victoria's Jubilee Institute for Nurses, shows that on October 31st, 1903, the Scottish Council were responsible for the maintenance of 51 nurses and probationers. During the year 37 nurses were engaged on the completion of their training at the Home in Glasgow by local committees, and 13 new branches were established. We note, with pleasure, that in some places the working classes make an organised effort to support Queen's nurses. At Prestonpans every miner makes a weekly contribution, and every fishing-boat an annual contribution; Kinneil Collieries, Bo'ness, entirely support a Queen's nurse for their own district; Kelty and Cowdenbeath nurses are supported from the proceeds of the Gothenburg public-house system. There are now 238 Queen's nurses in Scotland under 164 affiliated branches, of which 25 are attached to county associations. Although the excess of ordinary expenditure over ordinary receipts was £356, the financial outlook appears to have improved. But a still further number of regular subscribers is obviously needed.

### THE JAPANESE ARMY NURSING SERVICE.

IT is of the utmost importance that in the event of war between two great powers English nurses should not appear to take sides, and we therefore regret that the indiscreet course of the American nurses who, anticipating an outbreak of hostilities between Japan and Russia, offered their services to the Japanese Minister in the United States, has been commended in England. Whatever may be the private opinion of nurses, it is not within the scope of their professional duties to be partisans of either Japan or Russia, and only harm can be done to nursing by any movement that may tend to render them so. As a matter of fact, if unhappily Japan goes to war, it has sufficient nurses of its own. In our issue of April 25 last year, we published an account of the nurses of the Japan Red Cross Society. From this it may be seen that nearly a year ago there were more than 1,500 nurses already trained, and upwards of 600 still under training, who would be available for service in the event of war. Both in the conflict between Japan and China ten years ago, and in the North China troubles of 1900, the nurses of the Japan Red Cross Society vied with each other in reporting themselves ready for work; and as they join it, not in order to earn a living, but purely for the purpose of affording succour to military patients in time of need, there is no doubt that in any circumstance there would be an adequate rally. Japan has, in short, its own Army Nursing Service, which renders offers of foreign aid quite superfluous.

### THE MIDWIVES BOARD AND APPLICATIONS FOR CERTIFICATES.

AT a meeting of the Central Midwives Board, held at the board-room on December 17th, the Report of the Standing Committee as to the principles on which the Board should act in dealing with applications for recognition as approved institutions, or as approved teachers, was adopted. In accordance with the recommendations of the committee the Board resolved to accept the signature to Form V. of registered medical practitioners who are past or present holders of appointments to lying-in institutions or maternity charities; past or present examiners in midwifery to any examining board for a medical qualification; or lecturers on midwifery to institutions where pupil midwives or students of medicine are instructed. The Board decided to consider individually and on their own merits applications from registered medical practitioners not coming within these categories; and to postpone for the present all applications from teachers in Poor-law institutions, in view of the fact that almost all midwives exercising their calling in such institutions are, at the express instance of the Local Government Board, specifically exempted from the operation of Section E. of the Rules. Applications for recognition as approved institutions were granted to Queen Charlotte's Lying in Hospital, Liverpool Ladies' Charity and Lying in Hospital, Manchester Southern and Maternity Hospital, British Lying-in Hospital, London, Newcastle on-Tyne Lying-in Hospital, General Lying-in Hospital, Lambeth, Glasgow Maternity Hospital, Dundee Maternity Hospital, District Nursing Association, Cheltenham, and the Maternity Charity and District Nurses' Home, Plaistow. The Board resolved that a slip be appended to Form III. (certificate of attendance on cases) as follows:—"Note—Although a case of labour may be used for the instruction of more than one pupil, the case can only be counted to the credit of the one pupil to whom the actual delivery is entrusted." After consideration of applications for certificates the names of 270 women were passed under Section 2 of the Act, and ordered for entry on the roll. Of this total 65 claimed as holding the certificate of the Obstetrical Society of London, 5 that of the Rotunda Hospital, Dublin, 1 that of the Coombe Lying-in Hospital, Dublin, 1 that of the Liverpool Lying-in Hospital, 8 that of St. Mary's Hospital, Manchester, 2 that of the Salvation Army Maternity Home, and 188 were admitted as having been in *bona fide* practice for one year prior to July 31st, 1902. It would thus appear that the midwives trained in Irish hospitals are not, after all, to be shut out.

### NURSES AND PUBLIC WORSHIP.

THE Local Government Board have had imposed upon them by the Ecclesall Bierlow Guardians, at Sheffield, the task of deciding whether it is compulsory for Poor-law nurses to attend Church service on Sunday mornings. The question is not one of Church or Chapel. It has arisen in consequence of the superintendent nurse declaring that she objects, as a matter of conscience, to attend Church at all. We regret that any person choosing a vocation which necessarily brings her frequently in contact with those who are passing away to another world should object to attend Church at all. Nurses whose consciences impel them to repudiate religion have mistaken their calling. But we do not think that the Local Government Board will uphold the view that refusal to attend Church constitutes an act of insubordination. To talk about "compelling Nurse Pascoe to attend Church," as some of the Ecclesall Bierlow Guardians did at their meeting, is not likely to have a good effect upon anyone, especially as it appears that the alleged insubordination has been going on more or less for two years. The guardian who observed that if the Board had intended to require that Miss Pascoe should attend Church every Sunday they should have told her so when she first entered their service, indicated the course that should be pursued in future before a nurse is engaged.

### STATE REGISTRATION.

A special meeting of the general council of the Royal British Nurses' Association will be held at 10 Orchard Street, London, at 5 P.M. on Wednesday next, in order to consider a report from the executive committee with reference to the presentation of a Bill "For the State Registration of Trained Nurses" to the House of Commons. A special general meeting will be held at the rooms of the Medical Society of London, 11 Chandos Street, Cavendish Square, W., on Friday, January 8th, at 5 P.M., to consider a report from the general council with reference to the same Bill.

### NURSES' HOME AT THE CANCER HOSPITAL.

WE are glad to hear that a nurses' home for the staff of the Cancer Hospital is to be begun almost immediately. It is hoped that the home will be ready for occupation within twelve months. The site chosen is within the bounds of the garden, where a mulberry tree now stands. There will be provision for sitting and writing rooms, as well as for separate bedrooms, and all the day staff will be accommodated. The night nurses have already a special house. A portrait of herself by a former patient has been presented to the home by Miss Rogers.

### IMPROVEMENTS AT ROYAL HANTS COUNTY HOSPITAL.

RECENT structural alterations at the Royal Hants County Hospital, Winchester, have enabled the nurses to vacate their former small sitting-room and take possession of a much larger one, over-looking the gardens, and commanding a distant view of the City and St. Catherine's Hill. This was formerly a special ward. The red-curtained windows are lofty and mullioned; the floor is of walnut, partly covered by handsome squares of carpet. The furniture includes many easy chairs, a sofa, and a Chesterfield couch, a particularly beautiful Cromwell table, and an expensive Japanese screen. The room is heated by hot water coils, and has an open fire-place. Another improvement is a sick-room for the nursing staff, for whom no such separate accommodation has hitherto been provided. Both apartments are on the top floor, the sick room being well away from the wards. Additional cubicles have also been built; these are light and cheerful, well warmed by hot-water pipes, and comfortably furnished.

### THE NURSES OF BRISTOL CHILDREN'S HOSPITAL.

PRIZES and certificates to the nurses of the Bristol Royal Hospital for Sick Children and Women who came out well at the examinations in anatomy and physiology and medical nursing, following upon lectures by members of the Medical Board, were presented by the Duchess of Beaufort in the presence of a large company. The Duchess expressed the pleasure it gave her to perform the ceremony, and then presented the prizes as follows: Anatomy and physiology, second year nurses, Miss Moseley, prize; Miss Jarman and Miss Cooper, certificate of honourable mention. First year nurses, Miss Corbett and Miss White, prizes; Miss Lewis, Miss Thompson, and Miss Jefferys, certificate of honourable mention. Medical nursing, second year nurses, Miss Moseley, prize; Miss Jarman, certificate of honourable mention. First year nurses, Miss Corbett, prize; Miss Lewis and Miss White, certificate of honourable mention. Votes of thanks having been accorded to the lecturers for their services during the past session, Miss Mattick, the matron, proposed a vote of thanks to the Duchess of Beaufort. In congratulating the nurses who were successful as prize-winners and holders of certificates the Matron explained, by way of consolation to the non prize winners, that it did not necessarily follow that the best nurses were those who carried off the prizes. Very often the practical work in the ward, and tact and kindness in dealing with patients, carried more weight with the matron, and she had much reason to be pleased with many of the nurses to whom no prize had been awarded, a very high standard having been reached by all.

### THE MATRON OF BRECHIN INFIRMARY.

A special meeting of the directors of Brechin Infirmary has been held for the purpose of dealing with the resignation of Miss Fraser, the matron. It appears that Miss Fraser does not approve of the new regulations which have been formulated by the committee. She thinks that they are impracticable, but as they have been almost unanimously adopted by the directors, it was decided to accept her resignation. An amendment that it be not accepted only met with the support of the mover.

### NURSING IN NORTHERN WORKHOUSE INFIRMARIES.

The nursing returns for last year for the northern district, consisting of Northumberland, Durham, the North Riding of Yorkshire, and part of Cumberland, issued by Mr. James Lowry, general inspector of the Local Government Board, has features which are not by any means satisfactory. The number of nurses on duty day and night is 153, or one nurse to 18 patients. It is true that there are 208 pauper attendants, but the sooner the latter are dispensed with the better. Mr. Lowry's details reveal the fact that some counties are worse offenders in this respect than others. Thus, the average number in Northumberland of patients to a nurse is 18, while the pauper attendants engaged in the union are 47. In Durham the numbers are 22 patients to a nurse, and there are 107 pauper attendants. In the North Riding of Yorkshire there are 14 patients to a nurse and 50 pauper attendants, and in Cumberland 15 patients to a nurse and four pauper attendants. Mr. Lowry has for some time past been urging the boards of guardians in his district to augment the strength of their nursing staffs, and his returns for the year more than justify insistence on the necessity for immediate action.

### PAINFUL CHARGE AGAINST A NURSE.

On Monday, at Bow Street Police Court, Miss Mary Irwin Shaw, a trained nurse, was committed for trial on charges of theft. She is accused of stealing several articles of clothing from other nurses staying, like herself, at the Nurses' Hostel in Francis Street. When her box was opened in the presence of Miss Wood, the superintendent, property belonging to four other nurses was found in it. Suspicion attached to Miss Shaw in consequence of several cases of theft at the hostel lately, and she admits that two of the charges are correct.

### THE NURSES OF GREENWICH WORKHOUSE INFIRMARY.

The Infirmary Committee of the Greenwich Board of Guardians have reported that as the result of the recent examination of nurses, four out of seven seniors were approved, namely, Nurses Flood, Sanders, Sandy, and Carter; and four out of five juniors, namely, Nurses Spriggs, Kennedy, Chapman, and Newton. Dr. J. H. Bryant, who conducted the examination, pointed out that in the selection of probationers a good preliminary education was essential.

### PROPOSED NEW DEPARTURE AT TRURO.

It was suggested at the annual meeting of the Truro District Nursing Association that a private branch should be established. The reason given was that the present financial position is precarious, but as the receipts last year covered the expenditure, the committee will doubtless pause before they take a step which they might have reason to regret. If more money is wanted, more subscribers should be secured The number of persons in Truro willing to contribute a yearly sum must surely exceed 229, and £170 a year, the maximum amount said to be required, ought to be obtained without much difficulty in the cathedral city. During an outbreak of typhoid fever, the nurses rendered the most valuable service.

### AMATEUR NURSES AND THE FINSEN LAMP.

Nurses not being available to work the Finsen lamp at the large hospital at Berne, an appeal was made to ladies of the city and neighbourhood. There has been a ready response and a sufficient number now attend regularly and apply the lenses. As nurses who have done the work know, it is by no means a light undertaking to keep one fixed position with unswerving attention for over an hour. But the doctors seem quite satisfied with their amateur assistants. No nurse is present; the only person not absolutely an amateur, besides the doctor, is a lady secretary, who has been taught that special work and who conducts the correspondence connected with it. It is stated that there have been some most satisfactory results from the treatment. But we are glad that in England trained nurses are available.

### OUR CHRISTMAS DISTRIBUTION.

Since we went to press last week we have received another parcel of clothing for our Christmas Distribution from Nurses Coutts and Swinburn, which we have forwarded to the matron of Guy's Hospital. A parcel which appeared to have been sent anonymously was from Miss Alice Seaford.

### The Nursing Outlook.

"From magnanimity, all fear above";
From nobler recompense, above applause,
Which owes to man's short outlook all its charm."

### NEW YEAR THOUGHTS.

THERE are probably few of us who do not approach the New Year without backward glances of regret and forward looks of hope. After the excitement of Christmas comes a time of pause, of contemplation; a time in which we should all gather up our strength and so start towards our future full of good resolutions and the desire to achieve better work.

It is well sometimes to close the door on the past and start afresh; to put away all remembrance of old troubles and quarrels and the irritation they caused, and to go forth with a clear and sunny heart ready to receive all faith and hope and love. The life of a nurse must necessarily be trying if it is lived out to the full, and if the nature has not hardened; but all its hardships have their smooth side if only we would follow the example of the poet:—

"The inner side of every cloud
Is bright and shining;
I therefore turn my clouds about,
And always wear them inside out,
To show the lining."

And there are certain practical ways of securing immunity from worry which every nurse knows, but which few nurses follow. Take the question of rest and fresh air for example; how often a day or two in the quiet of the country surrounded by the beauty of the fields and woods would put happiness into a tired and peevish heart, if only the nurse would leave her lodgings and go in search of the sunshine. Or even one day spent in walking across Hampstead Heath or Richmond Park, feeling the winter wind blow away the cobwebs, and enjoying a rough lunch at some little baker's shop, what a change it can give to the tone of one's thoughts! So let us decide in the New Year to constantly seek the fresh air cure.

Secondly, there is the worry of advancing old age which some of us have to face. We shall not be able to work much longer, and what have we to look forward to in the future? Happy are those who began young to put into the Royal National Pension Fund for Nurses; they know exactly what they have to put by yearly, they know exactly what they can draw in the future—they need not fret. The old-fashioned theory of thrift, of saving the pennies and letting the pounds take care of themselves, is too petty and too constantly needing personal thought to fit in with the general unselfishness of a nurse's life. It is far better for her to have some definite payment to make at stated times, and then be free to think no more on the question. It is wonderful how comfortable and cheerful one can be on a little income, so long as that income is quite secure. In Italy, for instance, one can live comfortably on £50 a year, with the best of art and the most brilliant of sunshine thrown in for nothing! Therefore, let us decide to at once make provision for our future; at once join the Pension Fund if we have not done so, and then give no further thought to the morrow.

Then, thirdly, the greatest hope for achieving happiness, for performing good work in the New Year, lies in securing a heart which is in perfect oneness with the will of God. It is said to have been Jowett who described the Toynbee Hall young man as "ever eager to run to the rescue of God," and some nurses become so oppressed with the suffering they witness that they also get into that morbid state when they feel as though the burden of the world's unsatisfactoriness lay on their shoulders; and instead of doing their best with the circumstances in which they find themselves, they try to alter the course of nature, and throw themselves into conflict with natural things. Now this is quite useless and only leads to a petty and a peevish state. At the bottom of all religion and all philosophy we find this fact of the acceptance of life as it is. Says Professor Caird: "Oneness of mind and will with the divine mind and will is not the future hope and aim of religion, but its very beginning and birth in the soul. To enter on the religious life is to terminate the struggle." And compare the cry—"Not my will but Thine be done," with Socrates' last words—"O Crito, if it seem good to the gods so let it be." If we would secure tranquil-mindedness through all the coming year, we must found it on surrender of our own idea of what is right and just for us and others, and then all our paths will be found freshly smoothed. This also is a most practical, and not only a moral consideration, for we know now the value of "suggestion" as a cure agent, and we know that the nervous and unhealthy-minded nurse must have a bad influence on her patient. Men and women are all too ready to fear, to dread disease and death, and hence their natures become cramped and depressed, and their lives miserable; but mind-cure develops a system of mental hygiene which bids us think on health, vigour, and happiness, bids us ever seek the optimistic view: the force of personal faith, enthusiasm, and example are prime suggestive agencies for the recovery and cheerfulness of our patients. We understand nowadays how the martyrs sang at the stake, and faced the lions unmoved. They had secured inward peace by throwing aside all burdens, and the joy that shone on their faces, the love that was in their souls, might well have healing power. By these, or by other paths, we sincerely hope that our readers, one and all, may attain

A HAPPY NEW YEAR.

### Lectures Upon the Nursing of Mental Diseases.

By Robert Jones, M.D.Lond., B.S., F.R.C.S.Eng., M.R.C.P.Lond., Resident Physician and Superintendent of the London County Asylum, Claybury.

### LECTURE III.

The highest form of mental action results from abstract thoughts. When they lead persons to do anything, they are called motives, and when these suggestions of conduct are so clear that we are able to deliberate upon them, acting only after some reflection, they give a sense that the action springs from our own "will" or choice. Hence the phrase "free-will." No action can take place without the appropriate thought to suggest that action. With certain affections of the cortex of the brain—destroying the memory of how words sound, how they appear when written, or feel to the tongue, lips, or larynx when uttered—speech becomes impossible, although there is no loss of any movement connected with the vocal organs or speech. Such a condition occurs in what is called "word-deafness," "word-blindness," or "sensory aphasia," a condition which causes irritability and loss of control, so that the subjects thereof become not infrequently the inmates of our asylums.

Action and reaction are always equal and contrary. After a certain time of activity, rest is required. The period during which the brain rests is when sleep comes on. All the bodily functions are lowered during sleep, the pulse beats slower, and the temperature is reduced. When all sensations cease to enter the mind sleep is obtained. Sleep is favoured by the feeling of fatigue, which also is a condition of lowered action. Sleep is as necessary as food, and especially is it necessary when the brain is overwrought and agitation or restlessness has brought on a disturbance of consciousness, and the functions of the brain are disturbed. Sleep is soundest during the first hour or two. When the brain is in a condition partly awake and partly asleep then is the state known as dreaming prone to occur. In dreaming, some external sensation starts a train of ideas and these ideas are not under the control of the higher powers of the mind. Dreams are like delusions, except that during wakefulness and on an appeal to reason their unreality is apparent. Dreams are very rapid, a long dream full of adventure often taking only a few minutes. When the dreamer walks in his sleep he is a somnambulist, the balance of the movements being maintained reflexly. The great importance of sleep will be brought to our notice later.

We have referred to the *instincts*, to *reflex*, and to *automatic* actions. There is another psychological division which greatly influences the mind of man, viz., the *appetites*. In so far as mental unsoundness is concerned with some departure from the normal in the sphere of thought, emotion, or reasonable conduct, such departure being by way of defect, as in idiots, or irregularity (alteration, perversion, or reversal of healthy actions) as in the insane, it is necessary that we should refer to some of these mental activities somewhat in detail.

*Instinct* has been defined as "uneducated ability." Man and animals have found the performance of certain actions appropriate and useful for the propagation and prolongation of their species, and it is accepted that any intelligent adaptations for this purpose made by the individuals of one generation "sets the direction," as it is called, in those developed afterwards, and this "direction" in the nervous system tends to the performance of these actions quite independently of learning. This "setting the direction" has been termed "organic selection," but we are not concerned with the origin of the instincts and shall only refer to the fact. Man has grown to his present state by gradual stages, and not by sudden leaps or breaks; what we see in him we also see in many of the lower animals. Growth takes place under definite laws, and this growth is particularly affected by man's surroundings, which, so to speak, "condition" the physical and social nature of races of men as well as of individuals. The habits and customs of coloured people are totally distinct from those of white races. This influence of the "environment" is an important factor in the estimation of mental unsoundness. The age, the period in which a person lives, his *status* in society, his employment, education, and mode of life, and his relation to his surroundings, are all necessary factors when considering insanity. Conduct which would be highly proper and suitable to a person on one occasion would be madness in another on other occasions. If a clergyman were to shout comic songs from the pulpit, or a judge were to play with toys or to amuse himself with bits of broken china, or a shopkeeper were to jump over his counter instead of serving his customer, such conduct would be unsuitable and not only reprehensible, but it would in all probability be a sign of insanity, although under other circumstances such conduct would call for no comment. Instinct is essentially concerned with the preservation of the life of the individual self or directed towards its reproduction and continuation. An instinct is adaptive in its nature, it is relatively complex, and is inherited. Instinctive actions are witnessed in the maternal attentions of mankind and animals; pecking for food by the young fowl just emerged from the shell, sucking in the infant, swimming in the young water-bird, and the migrating actions of adult birds and mammals, are all further examples of instinct. The preservation of life and the quest of pleasure are human instincts. As to pleasure, it is an almost invariable rule that states of the body that are favourable to growth are pleasurable, whilst those that depress vital activity are painful. In insanity there is often a marked perversion of the instincts, and a reversal of the instinct of self-preservation is exemplified in the refusal of food and the various suicidal tendencies that are met with in many cases of acute melancholia or mental depression. Disorders of the parental instinct are frequently seen in the fiendish rage of destruction directed against the new-born offspring in the insanity of childbirth.

*Appetites.*—These are a special class of sensations, defined as uneasy feelings caused by the recurring wants or necessities of the organic life. They are sleep, exercise, repose, thirst, hunger, and the feeling of sex. These several appetites serve as motives for the will to act, as is exemplified when sleep or hunger are thwarted, but the appetites are not much controlled by the will. Appetite is not impulse, for it is well-defined and especially seated in different organs. Appetite is distinguished from instinct in that it shows itself at first in connection with the life of the organism itself and does not wait for an external stimulus. The movements by which appetite is qualified are mostly reflex and instinctive. The child has the instinct of sucking to gratify the appetite for food. In insanity there may be very considerable disturbances of these various sensations, to which we shall refer later.

*Reflex Actions* are a certain class of living actions maintained through a centre, which acts so that certain movements result in answer to certain stimuli or excitants. Reflex actions are non-voluntary and arise without any feeling, intention, or exercise of the will. Although the stimulus may reach the brain, and although it takes place without the intervention of the will, or even the participation of consciousness, it does not follow that the person is unconscious of it; he may be fully conscious of the whole reflex act. The commonest form of reflex action is muscular contraction following directly upon an irritation of the skin, a blinking of the eyelids when anything approaches the eye, the knee-jerk, etc., but there are many other forms of reflex action. The flow of saliva at the sight of food is one, flushing of the face at an emotion of joy, and pallor at the thought of fear, are others; also contraction of the pupil when a bright light is brought before the eye. Coughing and sneezing are further reflex acts. Reflex actions are thus not only sensori-motor—*i.e.* result in muscular contraction, but are also glandular, vascular, and ideo-motor. Reflex actions are not adaptive, they are in addition simple and not complex. Reflex action may be very considerably disturbed in insanity.

*Automatic actions* should be distinguished from reflex actions as the latter are distinguished from instincts. These are actions so often performed that the organism has become used to them, and the attention is not engaged in their repetition. They are best defined as a series of acts caused by a series of stimuli which do not necessarily rouse the attention. They are acquired, and not congenital or inborn, as are the instincts. They are either primary, as in the case of respiration, or secondary, as in the more elaborated acquired movements already referred to, such as walking or playing the piano, which can occur without thought or without engaging the attention.

*Habit and Conduct.*—The term *habit* is used in regard to a mental function, which being performed repeatedly tends through familiarity to be performed with facility. The law of habit is that any function becomes organised by repeated efforts. It is in this way that a function is acquired by the individual, and is sharply distinguished from an instinct which is physically inherited. The habitual routine of life becomes a species of appetite, and interference with this is exemplified in the mental breakdown of persons who after a busy and energetic career seek repose in retirement. *Conduct* is for our purpose the sum of an individual's actions, but authorities differ as to this meaning; some considering conduct to be the state of mind, or the intention; others deem it to be the outward act. Two phases of conduct especially concern us, viz., the conduct of a person towards him or herself, and that which refers to the environment, viz., to the surroundings or to society. Every self-respecting person is brought up to observe the decencies of life in regard to personal cleanliness and clothing. Man washes if he does not also bathe, and he wears his clothes to hide his nakedness—if not for warmth, appearance, and comfort. In regard to himself when in health, man also feeds and clothes himself with propriety. He is careful to select nourishing and palatable food, according to his means, and to take it at properly regulated intervals. He gives the necessary attention in the appointed place to the apparatus and organs of excretion. As to sleep, rest, and work, he is careful to observe the required periodicity, remembering that action and reaction are always equal and contrary. In his behaviour to others he returns the ordinary salutations implied in the politeness of the three C's—courtesy, ceremony, and convention. In insanity, however, a complete reversal sets in, and the various acquisitions which characterised his personality undergo modification, if they do not entirely disappear—those last attained being the first to go. His conduct may change, because he may regard food or clothing as being forbidden to him. He may undergo such extreme self-denial that he would starve if left alone. He will resist food with stubborn obstinacy, and refuse to be dressed or to take exercise; and he will surround himself with morbid and unnecessary restrictions. He may, on the other hand, display lavish prodigality, regardless of those dependent upon him, and he may assume fantastic attire or become quite self-neglectful. His habits in regard to personal cleanliness may become defective and even revolting. He may pick up rubbish and harbour dirt so as to be unfit to associate with others. As to his environment, he may exhibit a complete reversal of his previous conduct and avoid the society he formerly courted and appreciated, refuse to work, and show a complete distaste for all forms of exertion, leading a passive, dull or listless existence, or even take pleasure in giving trouble, becoming irritable and suspicious, destroying his clothing or property, and becoming quarrelsome and dangerous. The self-restraint of former days may change to aggressive excitement, aad he may take a violent dislike and hate to those nearest to him in ties of kinship and friendship. Such are, shortly, some of the perversions to be anticipated. Of such are the inmates of our asylums, and it needs strong qualities of head and heart to deal with these in a spirit of firmness and kindness. It is not too much to say, however, that good feeling, watchfulness, and tactfulness on the part of a mental nurse are capable of evolving order out of this chaos, and although the work is always trying and often arduous, women who are bright, intelligent, sensible, and sympathetic, not only can change the character and conduct of one patient, but also those of a whole ward, and there is no department of medicine and no illness in life in which previous experience and training on the part of a nurse are so valuable as in nursing a case of recent acute insanity. A good nurse in such a case is of incalculable benefit.

### Christmas in the Hospitals.

BY OUR OWN COMMISSIONERS.

LIKE other classes, hospital officials have their differences of opinion, and neither medical superintendents nor medical students always see eye to eye; while matrons, sisters, and nurses often look at their obligations from an individual point of view which is not necessarily identical. But when it is a question of the reputation of the institution to which they are attached they are united in a common cause. Certainly, whatever little jealousies or discords may be allowed to prevail at other periods, they are all thrown aside when that season of the year arrives which is devoted to the congenial work of endeavouring to make the sick and suffering feel that, though they are compelled to spend their Christmas away from home, they receive in hospital a measure of kindly attention that can only be the outcome of a general, as well as a genuine, desire to afford them every possible happiness. It is largely the unity of purpose which obtains among the members of the staff that secures for the patients the comforts, the entertainments, and the gifts that do so much to reconcile them to their detention in the wards. Each vies with the other to render the programme attractive, and every succeeding year there is a laudable ambition to introduce an element of novelty. There is, however, a limit even to the resources of the most ingenious minds. All that, in ordinary cases, can be done is to give an extra finish to the decorations; to choose the presents with greater consideration for the individual recipient; to arrange the entertainments which are most popular with the patients, even if they are more tiring to the performers; and to exhibit by drawing upon an ever-increasing fund of patience and tact, added sympathy for people who do not invariably merit it, yet because of their affliction have an irresistible claim to gracious and tender ministrations. This

### CHRISTMAS IN THE HOSPITALS—*Continued.*

unity of purpose is all the more remarkable and admirable because both the medical and nursing staffs are constantly changing some of their members. Yet the work of preparing for the Christmas festival always goes on. The spirit which has pervaded the hospital for years is soon caught by the new probationer, or the budding surgeon; hence the enthusiasm that is needed to maintain the standard. Nowhere, perhaps, is enthusiasm a more potent force than in the wards of one of our great charitable institutions, and there is no doubt that the secret of its prevalence is the contagion of personal example. No one can live for any length of time among those who desire to benefit their fellow-creatures without being infected by it, and thus at Christmas, when hearts are willing and hands are ready, the one object aimed at is to ensure the maximum of enjoyment for those who are not able to help themselves. The accounts of the representative festivities which follow, attest, once more, the completeness of the success that attends such labours, which is all the more welcome because they are untainted by selfishness.

### Guy's Hospital.

The annual treat organised for poor children of the Borough, including many out-patients, took place as usual at Guy's Hospital on Christmas Day. Tea, a magic-lantern, a cinematograph, a nigger troupe, Christmas tree toys and loaves of bread were provided for about seven hundred guests, and by four o'clock the air was thick with the beating of drums, the blowing of whistles and trumpets, and the voices of children, above which was heard the "Stand back" of the friendly policemen who assisted the medical officers, sisters and nurses in maintaining order when the time for departure arrived. The nigger troupe, composed of students in motley garb and armed with an enormous drum and uncouth wind instruments, was busily employed in visiting the wards from one o'clock until nearly seven, a very amusing farce forming part of the programme. This was entitled "The O'Course-'e-can Brothers," and was full of topical allusions. The *dramatis personæ* included Baron Bacillus, Captain Staphylococcus, a ruined Greek, and Ermyntruda, a noble lady, represented by Mr. Hughes. The printed programme was illustrated by a realistic scene at an operation, supposed to have taken place at Guy's between the years 302 and 309 B.C., in which a crowbar and sledge-hammer played an important part. The wards were very tastefully decorated with ivy screens and coloured lamps, and the windows overlooking the courtyards were aglow with lights throughout the evening.

### St. Bartholomew's Hospital.

Sounds of mirth and applause came from many wards surrounding the courtyard of St. Bartholomew's Hospital as dusk fell on Christmas afternoon, and coloured lamps in the windows lighted up the outside gloom and flashed in the water of the central fountain The patients of Rahere, the founder's ward, were entertained by an excellent gramaphone, and someone had found time to string together hundreds of holly leaves wherewith to hang festoons from one bed-head to another. Ivy and misletoe laid on white paper formed an effective decoration for fireplaces and screens, and many fairy lamps glowed among the dark green. A bran tub and an amusing Chinese conjurer were among the attractions, the latter visiting several of the wards in succession. Luke ward had songs from one of the sisters, who accompanied herself on the harp; and Charity was visited by another harpist. The work of entertaining was in the hands of each ward sister, who was assisted by members of the medical and surgical staff and by the nurses.

### The London Hospital.

It was about half-past three on Christmas morning when a two-year-old patient in one of the children's wards at the London Hospital was half awakened by the sound of carol singing. She was soon asleep again, but the impression remained, and in the morning she informed nurse that she had seen the angels in the night. And indeed the whole of Christmas Day was a new and wonderful experience to the little denizens of the London, the wards of which were transformed by festoons of evergreens, Chinese lanterns and fairy lamps, flags, and Christmas trees. Each of the four children's wards had its tree, the fruit of which, in the shape of dolls, trumpets and drums and countless other fascinating toys, was distributed early in the afternoon, so that the convalescents might be ready to visit the adult wards where various entertainments were being given. A printed programme, with a view of the extensive hospital buildings fronting Whitechapel Road, had been prepared, and thirteen troupes visited the wards, three of them giving no fewer than eight performances between 3.30 and 7 o'clock. The Prehistoric Band, in shaggy wigs and sheepskins, and the Scarlet Runners, scarlet as to legs and arms, with green calyx and peaked hood and shoes, were perhaps among the most effective, but the efforts of all were thoroughly appreciated. Mr. Seymour Dicker's troupe was first on the scene, and while waiting for the entertainment to begin, the male patients in Gloucester ward regaled each other with "The Queen of the Earth" and other popular songs. Smoking was allowed, the tobacco being provided by the medical staff.

### King's College.

The wards of King's College Hospital presented a very bright appearance on the occasion of the Boxing-day entertainment, when the patients were drawn up in beds and easy chairs in order to view the various performers. The "King's Kolossal Kollection of Kurious Kaged Kreatures, all Kreeping, Krouching, Kringing, Krawling, and Kareering," was announced by an extremely seedy-looking individual bearing sandwich boards, and was accompanied by a red-coated band. It met with an enthusiastic reception. The cleverly-contrived elephant, donkey, monkeys, poodle, and tortoise, all alive and mostly very active, required an amount of keeping under control which called forth all the energies of the gorgeously apparalled ring-master, while the escapades of two clowns and Sunny Jim quite brought the house down, and the failure of the last-mentioned to jump a 12-inch fence until he had been sustained by handfulls of "Force," appealed very successfully to the audience. Another popular performer was the Strong Man, whose protruding muscles suggested concealed bandages or other stuffing, and who lifted weights said to be enormous. Nor were the jolly tars who formed another company less appreciated. The man-o'-war, with funnel and blue waves complete, was manned by a sailor who played a portable organ, and songs and dances were given by four handy men who drew the ship from ward to ward. Great pleasure was given by the performers in the concert by nurses and students, a song with a tea-tray accompaniment being extremely popular. The wards were elaborately decorated, one—Todd Ward—being decked with winter scenes in the window-sills, and a realistic representation of the Matterhorn, with scattered chalets, and a string of Alpine climbers nearing the summit. Another ward was Japanese in character. There was a Christmas-tree for the children on the Tuesday in Christmas week.

### Charing Cross Hospital.

Everyone worked hard to make the wards of Charing Cross Hospital look pretty for Christmas Day, and with excellent result. Wreathing was quite a feature of this year's decorations, and the tables were, as usual, very effective, chrysanthemums and violets, trailing smilax, and coloured lamps being generally used. Presents, festive teas, and music was the order of the day, and during the afternoon there was a visit from Mr. George Grossmith, whose sketches were intensely appreciated. An itinerant concert company, organised by members of the staff, went from ward to ward in turn, and songs and violin solos by medical officers, nurses, and friends made an excellent programme, greatly enjoyed by patients and friends. Almost every song had an encore, and the chorus of "Polly Wolly Doodle" was heartily joined in, while wreaths of smoke rose from wherever the male patients congregated.

### Westminster Hospital.

The wards of Westminster Hospital were visited on Christmas morning by Father Christmas in the person of one of the staff, who was accompanied by a melodeon, and a negro boy who turned catherine wheels. Then came the Christmas dinners for all who were on full diet, and these were followed by a tree for the children, in Marie Celeste ward, and, later still, by merry little tea parties for nurses, patients and friends, and a board-room entertainment, when an excellent programme was gone through. This was attended by all the convalescent patients. All the wards were as usual very prettily decorated with evergreen, coloured lamp shades, one, Henry Hoare, being transformed into a rose garden for the time being. The bran tubs took many original shapes, and in Northumberland ward was seen an enormous black and white football, which on examination was found to contain a variety of toys and sweets for the lucky dippers. Adelaide ward had a huge basket; Matthew had a barrow; Arden appropriately grew one of its forest trees; and Percy contrived a pond stocked with ducks and frogs. All the ward tables were daintily decked with flowers, ferns, and smilax, among which dishes of sweets were cunningly disposed.

### Middlesex Hospital.

The wards of the Middlesex Hospital were prettily decorated for Christmas Day with flowers, evergreens, and silk shades over the electric lights, while small electric arcs were disposed among the table decorations. Some beautiful chrysanthemums were used in the chapel, and special services were conducted by the chaplain. Every one in the hospital received a present, and all those who were well enough were invited to the Christmas tree in the board room, while bran tubs were carried round by the lady probationers for those unable to be present. Turkeys and plum puddings were provided for dinner, the former the gift of the surgeons, several of whom gave great pleasure to the patients by helping to serve the dinner. From two to four o'clock the patients received their friends, and there was an "extra nice tea." On Monday and Tuesday in Christmas week there were trees in the children's wards, Percy and Princess May, when the effect of the toy-laden branches, lighted by strings of coloured electric arcs, was particularly pleasing.

### St. Mary's Hospital.

All the wards at St. Mary's Hospital, Paddington, were tastefully decorated, and the green shades of the electric lights over the beds had a pretty effect. Carols were sung by some of the nurses and doctors on Christmas eve on the front staircase and on Christmas morning in the chapel, when the service was fully choral. On Christmas morning every patient received a present; the men were supplied with tobacco and pipes and allowed to smoke. Ward teas were given in every ward, and the tables groaned with the abundance of good things. Each set of wards had a piano, and where the condition of the patients allowed of it, music and entertainments of various kinds went on unceasingly from 4 to 8 P.M., when "God save the King" was sung. One of the male patients, on being asked what kind of a day he had had, just lay back on his pillows and said, with a deep sigh of satisfaction, "A real, ripping one, marm, thank ye." The children in the children's medical ward were brimful of glee. In the morning each one found a big stocking hanging to the cot filled with nice things, the examination of which kept all quiet during the morning. Then came the dinner and the plum pudding, and after dinner the sister induced them to take a little sleep until it was time to get ready for tea. They watched all the preparations with eager eyes, those who were up tried to help the nurses to lay the cloth, arrange the flowers, and bring in the provisions. They looked pretty in their pink jackets as they crowded round the piano, especially when one of their own doctors played. Every effort was made by the nurses and doctors to make each child feel that Christmas Day was the grandest birthday ever kept.

### The Cancer Hospital.

Even from outside it was evident that a good old-fashioned Christmas was being enjoyed by the patients of the Cancer Hospital, for every window was outlined with coloured lamps, and sounds of music floated on the air. This being the last Christmas to be spent as matron by Miss Rogers, whose retirement takes effect early in the New Year, special efforts had been made by the nursing staff, and each ward, as well as staircases and hall, was elaborately decked with lamps, evergreens, and flowers. In one instance a table was turned into a miniature duck pond, with swans and water-lilies, and in all it was evident that much thought had been expended as well as an amount of fatiguing work. On Christmas Day a band of 'cellos and violins played carols the peal of tubular bells rang to service in the prettily decorated chapel, carols were sung by the nurses, pianos being kindly lent by various firms for each ward, and the patients had their friends to tea. On Boxing Day concerts were given, and the Misses Vicani danced Irish jigs in costume.

### West Ham Hospital.

A number of supporters of the West Ham hospital, among whom were the Bishop of Barking, Mr. J. Roberts, M.P., and members of the Borough Council, paid a visit to the wards on Christmas Eve, when presents from an enormous tree were distributed by Father and Mrs. Christmas. The entire hospital had assumed a holiday air, and every ward had a distinct character, the most striking, perhaps, being the women's ward. Not only the patients, but the sister and staff nurse were in keeping with the decorations, the former having their hair dressed in Japanese style, with large combs and pins, and the latter wearing full Japanese costume. Over each bed was an umbrella, a similar one, but much larger, depended from the centre of the ceiling, hung round with dolls and fans, and the tables were decked with chrysanthemums and peach-blossom. A string of lanterns were strung from the door of "Japland" to that of "Babyland," gay with pink and white ribbons and flowers. Here the Christmas-tree was placed. The two male wards were decorated, one with a ship of flowers drawn by doves, to represent the Voyage of Life, and the other with flocks of butterflies, the idea being the Flight of Ages. The patients wore buttonholes of holly or violets, and evidently greatly appreciated the efforts made to give them an enjoyable Christmas, those who owing to the number of cases in the hospital had to be content with a mattress on the floor seeming as cheerful as any. During Christmas week two entertainments were given, a theatrical performance by the resident staff of doctors, secretary, and nurses, and a conjuring exhibition.

(*To be concluded next week.*)

### Everybody's Opinion.

### DISCIPLINE AT CHARING CROSS HOSPITAL.

MISS EMILY C. HALLIDAY, MATRON OF THE VICTORIA HOSPITAL FOR SICK CHILDREN, HULL, writes: Kindly allow me space in your paper to state that during the seven years I worked in Charing Cross Hospital, as probationer, nurse, and sister, the discipline was of a high standard. Late leave, 10, or theatre leave, 11.30, was never expected as a right once a week, but only granted as a privilege by the lady superintendent. The limit of time for those members of the nursing staff who were off duty in the evening being 8 P.M.

### DARLINGTON HOSPITAL.

THE LADY SUPERINTENDENT OF DARLINGTON HOSPITAL writes: May I call your attention to a slight error which occurs in your account of the nursing in the above hospital? The account says, "355 operations . . . since its opening," but this should have been "since the beginning of this year."

### THE ANGLO-AMERICAN NURSING HOME AT ROME

MISS E. C. VANSITTART, member of the first elected Committee of Management, writes from 36A Via Palestoi, Rome: Though I am totally unaware who "A Resident in Rome" is, I entirely agree with Sir Rennell Rodd in his desire that facts should be verified before assertions are made in public. I would therefore draw his attention to a few misstatements in his own letter in THE HOSPITAL of December 12th, 1903. "Nor at any time since the home has been opened has a free-bed patient been refused." How about the case of Mr. ——? A letter from the hon. secretary of the Anglo-American Nursing Home (the accuracy of which can easily be verified by reference to his letter-book) lies before me. It is dated June 13th, 1902, and contains this statement: "It is just possible that, owing to the lateness of the season, the committee may not be able to admit new patients." This free-bed patient was refused; and on or about June 16th, 1902, the matron of the home left for England, and the home was closed, though in the committee's report for 1902, at paragraph 2 on page 2, it is stated that the home "was closed from July to October. The letter of "A Nurse" appeared in THE HOSPITAL of October 17th, not of November 17th. If the statements of "A Nurse"—"I, together with five nurses who have returned"—are equivalent (according to Sir Rennell Rodd) to "accepted engagements to return," and not that they were already in Rome at the date of "A Nurse's" letter, which must presumably have been written about October 10th, perhaps we are to understand that "three arrived in Rome on October 1st and a fourth on the following day," means that nurses had accepted engagements to return on those dates. The quibble is apparent, but possibly Sir Rennell Rodd is like "Alice in Wonderland" who thought that meaning what she said was the same thing as saying what she meant. These inaccuracies ought once more to prove to the committee, and their latest champion, the secretary of His Majesty's embassy in Rome, the desirability of ensuring accuracy by means of an impartial investigation as was proposed by the "disentient minority," amongst whom I number myself, at the general meeting of subscribers to the home, held on December 10, 1902, but refused by the "verdict of the majority."

### Death in Our Ranks.

THE death of Sister Ellen Ringroe from phthisis occurred at Rosslyn, Castlemain Road, Bournemouth, on December 22nd. Miss Ringroe was trained at the South Devon Hospital, Plymouth, and was for some years staff nurse and sister at St. George's-in-the-East Workhouse Infirmary, after which she was appointed superintendent nurse at the Union Infirmary, Christchurch. This post she was obliged to relinquish last February in consequence of illness.

### Appointments.

EALING ISOLATION HOSPITAL.—Miss A. E. Knox has been appointed matron. She was trained at Willesden, Sittingbourne, and Ealing Isolation Hospitals.

FULHAM INFIRMARY.—Miss Margaret Birkett has been appointed sister. She was trained at Middlesbrough Union Infirmary, and has since been sister of maternity wards at Selby Oak Infirmary.

MOUNT VERNON HOSPITAL FOR CONSUMPTION AND DISEASES OF THE CHEST, HAMPSTEAD AND NORTHWOOD, MIDDLESEX.—Miss Florence M. Swain has been appointed matron of the Country Branch Hospital at Northwood, now nearing completion. She was trained at St. Mary's Hospital, Paddington, and at the Bristol General Hospital, was staff nurse at the City of London Chest Hospital, Victoria Park, E., sister of the women's medical, subsequently senior sister of the men's surgical and accident wards, East Suffolk Hospital, Ipswich. She has since been senior sister at the Mount Vernon Hospital, Hampstead. She holds the City of London Hospital lying-in certificate.

NEW SOMERSET HOSPITAL, CAPETOWN.—Miss J. C. Child has been appointed matron. She was trained at St. Thomas's Hospital, London, and has since been sister at the Sussex County Hospital, Brighton, and matron of Lewes Hospital. She was chosen for active service in the Græco-Turkish war, and has been sister in Kimberley Hospital and matron of the Memorial Hospital, Bulawayo.

ROYAL HOSPITAL FOR CHILDREN, BRISTOL.—Miss L. Dryden has been appointed assistant matron. She was trained at St. George's Hospital, London, and the Children's Hospital, Birmingham. She has since been night sister at the Infirmary and Dispensary, Bolton.

WIMBLEDON ISOLATION HOSPITAL.—Miss Alice King has been appointed day sister, and Miss Mary Newton night sister. Miss King was trained at the Royal Hospital, Portsmouth, and subsequently held the posts of night sister at Hull Sanatorium, charge nurse at the Brook Hospital, Shooters Hill, sister at the Royal Orthopædic Hospital, London, district nurse at Chatham, Birmingham, and Stepney, and for two years matron of the Cottage Hospital, Tenbury. Miss Newton was trained at the Hull Royal Infirmary and the South-Western Hospital of the Metropolitan Asylum Board, Stockwell. She has since been charge nurse and temporary night superintendent at the Brook Hospital, Shooters Hill.

### Presentations.

MILTON HOSPITAL, PORTSMOUTH.—Mrs. M. A. Antram, matron of the Milton Hospital, has been presented on her retirement with a handsome clock given by the sisters and nursing staff as a mark of esteem and regard in which she is held by them. Alderman A. Leon Emanuel in making the presentation, said that during the 18 years in which Mrs. Antram had held the position of matron she had carried out her duties exceedingly well. Since her appointment nearly 9,000 patients had been under her care and she had not only been kind to them and all the staff under her charge but by her strict personal supervision and economy had been the means of saving hundreds of pounds to the ratepayers.

CANCER HOSPITAL, LONDON.—An illuminated address has been presented to Miss Rogers, the retiring matron of the Cancer Hospital, Fulham Road, London, by the members of the surgical staff; two silver reading-lamps by the committee and office staff; a gilt clock with enamelled face by the chaplain, house surgeons and nursing staff; and a set of ivory brushes with silver initials by the domestics.

NURSING INSTITUTE, BLACKHEATH.—Miss Duncan, Principal of the Blackheath Nursing Institute has received from her nursing staff a very handsome clock.

### Echoes from the Outside World.

### Movements of Royalty.

THE King and Queen, the Prince and Princess of Wales, and other members of the Royal family spent Christmas at Sandringham quietly. The quantity of beef given away by their Majesties on the estate amounted to about two tons. The Queen also distributed a great number of plum puddings and mince pies. It is announced that the King and Queen hope it may be possible for them to pay a further visit to Ireland during the course of 1904. The Princess of Wales has sent the Bishop of Stepney £25, with a request that the money may be handed to the Lord Mayor as a contribution to the fund about to be raised at the Mansion House for the relief of the unemployed, and as an evidence of the Princess's sympathy with the prevailing distress.

### The Forthcoming Royal Marriage.

IT is officially announced that the wedding of Princess Alice of Albany with Prince Alexander of Teck will take place at St. George's Chapel, Windsor, on Wednesday, February 10th. The King and Queen will be present and many other members of the Royal family are expected to attend. Others who have been invited are the King and Queen of Wurtemburg and the three sisters of the Duchess of Albany, namely, Queen Emma of the Netherlands, Princess Pauline of Bentheim-Steinfurt, and Princess Elizabeth of Erbach Schonberg. A feature of the wedding will be a group of little bridesmaids, selected from the children of Royal parents. The marriage of the parents of the bride-elect was solemnised in St. George's Chapel twenty-two years ago in April next.

### Fighting in Somaliland.

ON Christmas Day news reached the War Office that on December 21st there was fighting in Somaliland, the enemy sustaining severe losses. Our mounted troops surprised 2,000 Dervishes in Jidballi, 38 miles to the east of Badevein. The engagement lasted three hours, and the enemy's casualties are said to have been 80 killed and 100 wounded. Our losses are reported to have been two men of the British Mounted Infantry wounded, and one man missing; two Tribal Horse killed, and two wounded.

### Royal Patriotic Fund.

THE King has approved the appointment of the following as members of the Royal Patriotic Fund Corporation under the provisions of the Patriotic Fund Reorganisation Act, 1903:—The Duke of Connaught, President; Lord Edmund Talbot M.P.; Sir James Bell, Bart., a Glasgow shipowner; Sir Francis Mowatt, G.C.B., ex-Permanent Secretary at the Treasury; Lieutenant-General Sir T. Kelly-Kenny, K.C.B., Adjutant-General to the Forces; Sir K. D. Awdry, K.C.B., Accountant-General to the Navy; Colonel Sir James Gildea, C.V.O., C.B., of the Soldiers and Sailors Families' Association; Mr. F. T. Marzials, C.B., Accountant-General of the Army; Mr. W. Hayes Fisher, M.P.; Mr. H. E. Kearley, M.P.; Mr. D. J. Shackleton, M.P.; and Mr. C. H. R. Stansfield.

### Railway Disasters in America.

LAST week a terrible railway accident occurred to an eastward-bound express train on the Baltimore and Ohio Railroad. The train ran into a pile of switch timbers. The baggage-master of the express, although he was injured, saved an approaching westward-bound train by setting fire to his coat as a signal, and then fainted away. Sixty-three passengers are reported to be killed, and some of the injured, who were taken to the hospital at Connellsville, are not expected to recover. The dead were robbed by thieves until the police arrived. On Christmas Day another disastrous collision occurred between two passenger trains on the Père Marquette railway, near East Paris, Michigan. The accident is attributed to the high wind extinguishing the red signal indicating that one of the trains should stop at the last station it passed through and await orders, resulted in the loss of 18 lives and injuries to 31 persons.

### Another Accident on Scawfell.

IT is only three months since four tourists lost their lives on Scawfell. On Boxing-day another climber—and strangely enough one who in his capacity of reporter had supplied the papers with the details of the last catastrophe—met with his death on the same mountain. Mr. A. Goodall, of Keswick, and Mr. F. Botterill, of Leeds, had safely attained the cairn on the summit of Scawfell, and admired the beautiful sunset. In returning Mr. Goodall descended first, as he said "he wished to gain a little more experience in glissading," taking with him the only ice axe. In some manner he lost his balance, and falling 400 feet he struck against some rocks and was killed instantly. His companion, who had been a horrified onlooker, had then to attempt the hazardous task which had been so fatal to his friend. Fortunately he found the ice axe which Mr. Goodall had dropped during his descent, and after cutting his way down the ice, step by step, he reached a place of safety after three hours' laborious exertion.

### Death of Mr. George Gissing.

THE decease of Mr. Henry Seton Merriman has been quickly followed by that of Mr. George Gissing, another talented and painstaking novelist. Mr. Gissing died on Monday at St. Jean de Luz at the early age of 46. A native of Wakefield, he was educated at Owens College, Manchester, and first came into note in 1884 as the author of "The Unclassed." In 1891 he published "New Grub Street," which firmly established his reputation, and among his later works were "In the Year of Jubilee," "The Odd Women," and "By the Ionian Sea." His last book is "The Private Papers of Henry Ryecroft," and many of his readers affirm that it is also his best.

### Christmas Entertainments.

DRURY LANE THEATRE, by reason of the gorgeousness of its spectacles, usually ranks first among the pantomimes. "Humpty Dumpty" is the title given to the entertainment this year, but except that the familiar figure is seen on the top of a castle, is shot down and smashed, he is virtually out of the running. Mr. Dan Leno has returned to the stage after his recent illness. "The Anemone's Retreat" and the "City of Coral" are two especially beautiful scenes. "The Water Babies" at the Garrick Theatre in the afternoon is a revival from last year, and it is such a beautiful allegory, so well staged, so admirably acted, and so altogether charming, that no child nor grown-up person should miss it. In the evening "The Cricket on the Hearth" holds the boards. At the Court Theatre "Snowdrop and the Seven Little Men" is entirely played by children, the wee Fairy Queen being represented by Miss Geraldine Watson, who appears to be about eight years of age. The Prince is Mr. Walter Creighton, son of the late Bishop of London. "Snowdrop" is preceded by an adaptation of "Brer Fox and Brer Rabbit." At the New Theatre "Alice through the Looking Glass" is certain to attract those who admire Lewis Carroll's quaint fun, and "Little Hans Andersen" is a rival attraction at the Adelphi. The Hippodrome has an elephantine pantomime, and the grand zoological parade deserves special praise. The Crystal Palace boasts of both a circus and a pantomime to amuse the youngsters.

### Notes and Queries.

**FOR REGULATIONS SEE PAGE 135.**

*Maternity Fees.*

(119) A maternity nurse is engaged for a case on December 16. Unable to attend on December 12, she sends a substitute as arranged previously with the lady. After two days, though the doctor was satisfied, the lady refused to allow the substitute to continue the case, and insisted on the first nurse taking her place. Can the substitute claim for the month ?—*Nurse M.*

The substitute must arrange for compensation with the first nurse, the lady is only bound to pay the £6 6s.

*Midwifery,*

(120) I am anxious to train in midwifery, and would be glad if you would kindly tell me what are the nece sary qualifications required by the Central Midwives Board.—*Miss E.*

Write to the Secretary, Central Midwives Board, 6 Suffolk Street, Pall Mall, S.W.

*Hospital Training.*

(121) How shall I start in order to train as a hospital nurse ? I am 20½ years old, domesticated, tall, and strong. I cannot afford to pay a premium, and should require a small salary and uniform to begin with.—*L. O. C.*

You are too young for a general hospital. But the East London Hospital for Children, Shadwell, E., or the Evelina Hospital for Sick Children, Southwark Bridge Road, S.E., might take you if you would like to start in a children's hospital. See also "The Nursing Profession : How and Where to Train."

Can anyone tell me where I could get good training with a small salary ? I have had 15 months' training, and I find that most matrons object to former training.—*Puzzled.*

Have you applied at the workhouse infirmaries ?

*Preparatory Training.*

(122) I am going as probationer to a large London hospital next year, and should be glad if you would kindly tell me what would be the best preparation for it ? It seems hardly worth while to enter a small hospital, as I shall learn all that can be taught about nursing at my training school.—*Filomena.*

Get a good nursing handbook. Master elementary anatomy and physiology. If you have an opportunity learn dispensing and housewifery and sick cooking. As you live in Manchester, you might consult Miss Romley Wright, Manchester School of Domestic Economy, South Parade, St. Mary's Street, Deansgate.

*Claim.*

(123) Will you kindly tell me if a nurse, leaving her post at the end of 12 months, can claim a month's salary in lieu of the month's holiday due to her ?—*K. G. Z.*

It depends upon the wording of her agreement; but it is not probable that such a claim would be allowed.

*Door Plate.*

(124) Miss F., who is about to practise as a midwife amongst the poorer classes, would be glad to know what is the correct inscription to put on her door plate. Should it be "Miss F." or "Nurse F.," with the qualifications underneath ?

Either would be correct, but "Nurse" is better.

*Male Nurse.*

(125) Can you suggest an institution where a lad of 18 could be trained as male nurse, and is it usual to pay a small premium ? —*A. G.*

He is too young yet for the National Hospital for the Paralysed and Epileptic, Queen's Square, Bloomsbury, W.C., which so far as civil institutions are concerned, is the only training school for male nurses in the kingdom. The Royal Army Medical Corps trains male nurses at the Government hospitals.

**Standard Nursing Manuals.**

"The Nursing Profession : How and Where to Train." 2s. net ; 2s. 4d. post free.

"Nursing : Its Theory and Practice." (Revised Edition). 3s. 6d. post free.

"Elementary Anatomy and Surgery for Nurses." By William McAdam Eccles, M.D.Lond., M.B. 2s. 6d. post free.

"Elementary Physiology for Nurses." By C. F. Marshall, M.D., B.Sc., F.R.C.S. 2s. post free.

"Ophthalmic Nursing." By Sydney Stephenson, M.B., F.R.C.S., 3s. 6d. net ; 3s. 10d post free.

"Nursing in Diseases of the Throat, Nose, and Ear." By P. Macleod Yearsley, F.R.C.S.Eng., M.R.C.S 2s. 6d. post free.

"Fevers and Infectious Diseases." 1s. post free.

### For Reading to the Sick.

THE NEW YEAR.

FATHER, let me dedicate
All this year to Thee,
In whatever worldly state
Thou wilt have me be :
Not from sorrow, pain, or care
Freedom dare I claim ;
This alone shall be my prayer,
Glorify Thy Name.

If in mercy Thou wilt spare
Joys that yet are mine ;
If on life serene and fair,
Brighter rays may shine :
Let my glad heart, while it sings,
Thee in all proclaim,
And, whate'er the future brings,
Glorify Thy Name.

If Thou callest to the Cross,
And its shadow come,
Turning all my gain to loss,
Shrouding heart and home ;
Let me think how Thy dear Son
To His glory came,
And in deepest woe pray on,
"Glorify Thy Name."

*Rev. L. Tuttiatt.*

I begin to-day a new life, with new duties, new responsibilities, and I heartily pray that I may fulfil them in that Christian spirit which may in some measure atone for the imperfection in their performance. I can breathe no prayer for the present, but that a sense of our utter dependence on God may never leave me, and that He will in his mercy strengthen my faith and resign me to His will ; that whatsoever that will may require from me, be it in suffering or in joy, my comfort as well as my thankfulness may rest solely on Him.—*Maria Hare.*

Say not that the little things of thy life are common. God will cleanse them ; in the mosaic they will all be precious in their harmony with the completed whole.—*George Matheson.*

Thou hast been down in the valley of the shadow, and thou hast been looking up to the calm heavens to find thy God. Yet all the time, thy God has been beside thee in the valley, a sharer in the shadow of thy life. He was speaking in the voices that seemed to deny His presence ; He was manifested in the shades that appeared to veil His Form. He came to thee in the night that His glory might be concealed. He came to thee unaccompanied and unadorned, that He might know whether He were loved for Himself alone.—*George Matheson.*

Onward and upward still our way,
With the joy of progress from day to day ;
Nearer and nearer every year
To the visions and hopes most true and dear !
Children still of a Father's love,
Children still of a home above !
Thus we look back
Without a sigh, o'er the lengthening track.

